# TRAIL-Sensitizing Effects of Flavonoids in Cancer

**DOI:** 10.3390/ijms242316596

**Published:** 2023-11-22

**Authors:** Anderson Luiz-Ferreira, Teresa Pacifico, Álefe Cardoso Cruz, Federica Laudisi, Giovanni Monteleone, Carmine Stolfi

**Affiliations:** 1 Inflammatory Bowel Disease Research Laboratory, Department of Biological Sciences, Institute of Biotechnology, Federal University of Catalão (UFCAT), Catalão 75704020, GO, Brazil; alefecardoso83@gmail.com; 2 Department of Systems Medicine, University of Rome “Tor Vergata”, 00133 Rome, Italy; teresa.pacifico@uniroma2.it (T.P.); federica.laudisi@uniroma2.it (F.L.); gi.monteleone@med.uniroma2.it (G.M.)

**Keywords:** natural products, medicinal plants, polyphenols, apoptosis, tumor necrosis factor related apoptosis-inducing ligand, DR4, DR5, caspase-8, c-FLIP, survivin

## Abstract

Tumor necrosis factor-related apoptosis-inducing ligand (TRAIL) represents a promising anticancer agent, as it selectively induces apoptosis in transformed cells without altering the cellular machinery of healthy cells. Unfortunately, the presence of TRAIL resistance mechanisms in a variety of cancer types represents a major hurdle, thus limiting the use of TRAIL as a single agent. Accumulating studies have shown that TRAIL-mediated apoptosis can be facilitated in resistant tumors by combined treatment with antitumor agents, ranging from synthetic molecules to natural products. Among the latter, flavonoids, the most prevalent polyphenols in plants, have shown remarkable competence in improving TRAIL-driven apoptosis in resistant cell lines as well as tumor-bearing mice with minimal side effects. Here, we summarize the molecular mechanisms, such as the upregulation of death receptor (DR)4 and DR5 and downregulation of key anti-apoptotic proteins [e.g., cellular FLICE-inhibitory protein (c-FLIP), X-linked inhibitor of apoptosis protein (XIAP), survivin], underlying the TRAIL-sensitizing properties of different classes of flavonoids (e.g., flavones, flavonols, isoflavones, chalcones, prenylflavonoids). Finally, we discuss limitations, mainly related to bioavailability issues, and future perspectives regarding the clinical use of flavonoids as adjuvant agents in TRAIL-based therapies.

## 1. Introduction

Cancer is a heterogeneous disease caused by the irreversible alteration of cellular homeostasis and function. Cancer progression is the result of abnormal cell growth and differentiation, along with the loss of apoptotic function, which leads to the uncontrolled expansion of neoplastic cells and their spread into surrounding tissues and ultimately to a distant part of the body [1]. In recent decades, the burden of cancer incidence and mortality has rapidly increased worldwide, in parallel with both the growth and aging of the population, as well as changes in the prevalence and distribution of the main cancer-related risk factors (e.g., tobacco smoking, alcohol consumption, processed foods, environmental pollutants), some of which are linked to socioeconomic development [2].

According to World Health Organization (WHO) estimates, cancer is actually the first or second leading cause of death before the age of 70 years in 112 of 183 countries and ranks third or fourth in another 23 countries (Global Health Estimates 2020: deaths by cause, age, sex, by country and by region, 2000–2019. https://www.who.int/data/gho/data/themes/mortality-and-global-health-estimates/ghe-leading-causes-of-death, accessed on 22 March 2022), representing a major health problem and an important hurdle to increasing life expectancy [3].

In addition to surgical intervention, radiation therapy, chemotherapy, and immunotherapy represent the most common treatment options for cancer. Unfortunately, despite considerable advances in such strategies, the intrinsic and/or acquired resistance of cancer cells to therapies [4], the reduced or lack of efficacy of immunotherapeutics in the majority of cancer cases [5], and the side effects, even severe in some cases, of commonly used chemotherapeutics [6], represent major limitations. For these reasons, the identification of new anticancer approaches with increased efficacy against neoplastic cells and lower toxicity in normal tissues is highly desirable. As the resistance of tumor cells to programmed cell death is a hallmark of cancer and an essential feature of its development, a mainstay and goal of clinical oncology is the advancement of therapies aimed at triggering/restoring the process of cellular apoptosis in cancer cells [7].

In this review, we focus on the application of flavonoids to overcome tumor necrosis factor-related apoptosis-inducing ligand (TRAIL) resistance in cancer cells, highlight the underlying molecular mechanisms, and discuss potential future perspectives and drawbacks.

## 2. Apoptosis Signaling Pathways

**Figure 1:**
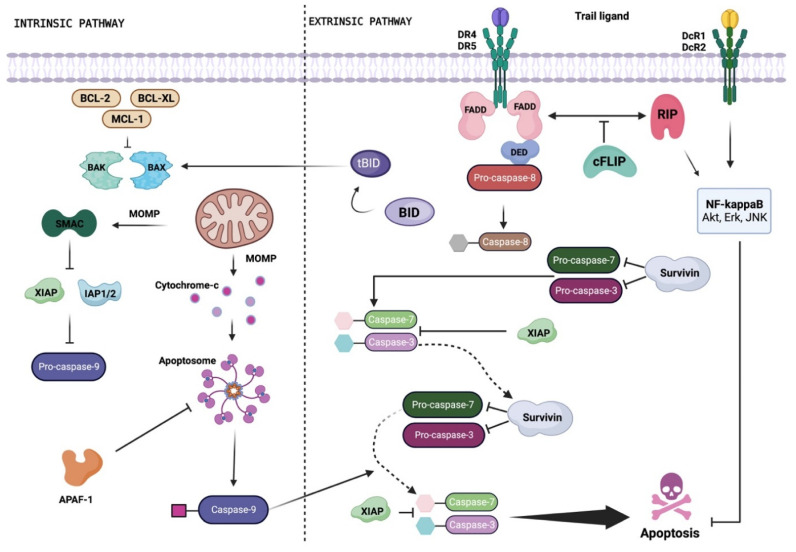
Schematic representation of the TRAIL signaling pathway. Depending on the cell type, TRAIL can induce apoptosis by activating the extrinsic or intrinsic pathway. Binding to DR4 and/or DR5 induces trimerization of the receptor, a prerequisite for the assembly of DISC. The Fas-associated death domain (FADD) adapter protein is recruited to the death domain of the TRAIL receptor through its own death domain, and FADD recruits pro-caspase-8 to the DISC through interaction with the death effector domain (DED), triggering proteolytic activation of caspase-8. Upon the release of DISC, active caspase-8 homodimers activate the effector caspase-3 to induce apoptosis. The activated caspase-8 activates the pro-apoptotic protein BID by proteolytic removal. Once activated (tBID), and with the participation of BAX and/or BAK, this protein induces mitochondrial outer membrane permeabilization (MOMP) and the subsequent release of cytochrome C, which forms an apoptosome with Apaf-1 and caspase-9, activating effector caspase-3. c-FLIP binds to caspase-8 and prevents its activation. Members of the IAP family (e.g., XIAP, survivin, IAP 1/2) negatively regulate caspase activation and can be inactivated by SMAC. Created with BioRender.com.

Apoptosis is a multi-step, multi-pathway cell death program that is inherent in every cell of the body. The apoptotic process is governed by multiple interconnected signaling cascades, referred to as the mitochondrial (or intrinsic) pathway and the death receptor (DR) (or extrinsic) pathway, triggered by several factors (e.g., cellular stress, DNA damage, and immune surveillance-related molecules). Both intrinsic and extrinsic pathways converge in the regulation of the caspase-dependent proteolysis of a plethora of cellular proteins, membrane blebbing, and the endonucleolytic cleavage of chromosomal DNA [7] (Figure 1).

In most mammalian cells, the critical requirements to activate the intrinsic apoptosis pathway are mitochondrial outer membrane permeabilization (MOMP) and the subsequent release of cytochrome c from the mitochondria into the cytoplasm, which leads to the formation of the apoptosome and caspase-3 activation (Figure 1). The release of cytochrome c from mitochondria is stimulated by pro-apoptotic members of the BCL-2 family (e.g., BAX, BAK) [8] and inhibited by anti-apoptotic members of the same family (e.g., BCL-2, BCL-XL, MCL-1) [8].

The second main apoptosis pathway, called the extrinsic pathway, is activated when specific ligands bind to cell membrane proteins known as DRs. Pro-apoptotic DRs encompass Fas—with the Fas ligand (FasL) representing the physiological ligand—tumor necrosis factor (TNF) receptors TNFR1 and TNFR2, and TNF-related apoptosis-inducing ligand (TRAIL, also known as APO2L and TNFSF10) receptors DR4 (also known as TRAIL-R1) and DR5 (also known as TRAIL-R2, Apo2, TRICK2, or KILLER) [9]. Upon ligand binding, activated DRs recruit adapter proteins, such as Fas-associated protein with death domain (FADD) and initiator caspases (e.g., caspase-8, caspase-10), to form the death-inducing signaling complex (DISC). The activation of initiator caspases is negatively regulated by the cellular FLICE-inhibitory protein (c-FLIP), a catalytically inactive caspase-8/-10 homologue that hinders the formation of DISC directly at the receptor level [9,10]. The trigger of initiator caspase-8 and -10 at DISC results in the activation of effector caspases (e.g., caspase-3, caspase-7) and ultimately apoptosis. In some cells, the activation of initiator caspases leads to BID cleavage and translocation to mitochondria, where it amplifies the apoptotic signal by contributing to the release of cytochrome c [9]. On the basis of the formation of the DISC and BID cleavage, cells can be classified into two distinct groups. So-called “type I” cells, which enable the fast and efficient formation of the DISC complex, promote the direct activation of effector caspases and, consequently, of the apoptotic event through the extrinsic pathway [9]. On the contrary, in so-called “type II” cells, the formation of the DISC complex is delayed and reduced, leaving these cells more dependent on the intrinsic apoptotic pathway [9].

### TRAIL as Potential Candidate for Cancer Treatment: Benefits and Drawbacks

As mentioned above, TRAIL is capable of inducing apoptosis by binding to DR4 or DR5. These DRs are type 1 transmembrane proteins that comprise an extracellular cysteine-rich domain (CRD), a transmembrane domain, and an intracellular domain that contains a death domain [9,11]. Consistent with the high sequence similarity between DR4 and DR5, the overall arrangement of the DR4-TRAIL complex (crystal structure available in Protein Data Bank, DOI: 10.2210/pdb5CIR/pdb) does not differ substantially from that of the DR5-TRAIL complex (crystal structure available in Protein Data Bank, DOI: 10.2210/pdb1D0G/pdb), although subtle differences are apparent [12]. Although both DRs are capable of triggering the same apoptotic signals, solution interaction studies have shown differences in the thermodynamics of the binding of DR4 or DR5 to TRAIL, with DR5 acting as the highest-affinity receptor [13]. However, TRAIL can bind to two additional cell membrane receptors, decoy receptor 1 (DcR1), also known as TRAIL-R3, and DcR2 (also known as TRAIL-R4), which do not induce apoptosis. In fact, although both decoy receptors contain extracellular CRDs, which exhibit significant homology to those of DR4 and DR5 (54–58% sequence identity), DcR2 contains a functionally inactive truncated cytoplasmic domain, whereas DcR1 is devoid of any transmembrane or cytoplasmic residues [14]. In addition to competing for TRAIL binding, DcRs can inhibit TRAIL-induced apoptosis by additional mechanisms. For example, DcR1 prevents the assembly of the death-inducing signaling complex (DISC) by titrating TRAIL within lipid rafts, whereas DcR2 is corecruited with DR5 within DISC, where it inhibits initiator caspase activation [15]. TRAIL can also bind to a secreted low-affinity receptor, termed osteoprotegerin, whose physiological importance remains, however, ambiguous [16]. Notably, TRAIL can selectively target neoplastic cells rather than normal cells, probably due to the significantly higher expression of DcRs in the latter [17]. This feature, together with the ability, unlike most chemotherapeutic drugs, to trigger the apoptosis of transformed cells independently of p53 [18], has suggested TRAIL as a powerful and safe candidate for cancer treatment. However, despite the promising results in experimental models, the efficacy of TRAIL in inducing apoptosis is hampered in a wide variety of cancer types due to the presence of intrinsic resistance mechanisms [19], representing a major limitation in the use of TRAIL-based therapies in the clinic and a current challenge. Although the molecular mechanisms underlying the decreased susceptibility of cancer cells to TRAIL-driven apoptosis have not been completely unraveled, the downregulation of DR4 and DR5, upregulation of DcR1 and DcR2 [11,20], and overexpression of c-FLIP and anti-apoptotic proteins belonging to both the Bcl-2 (for example, Bcl-2, Bcl-xL, MCL-1) and cellular inhibitor of apoptosis protein (IAP)—for example, X-linked inhibitor of apoptosis protein (XIAP), survivin—families are the most frequently proposed TRAIL resistance factors [11,20]. These observations, together with the notion that the defects mentioned above are reversible, have suggested the need for combinatorial strategies to overcome cancer cell resistance to TRAIL. Therefore, increasing research efforts have been aimed at identifying new and safe TRAIL-sensitizing agents [21].

## 3. Medicinal Plants and Flavonoids

Natural products encompass any compound or substance (presenting biological activity or not) derived from living organisms found in nature. Among natural products, those derived from plants have been recognized for their therapeutic properties for a very long time, being used in several parts of the world to treat a multitude of diseases [22,23,24,25]. Notably, half of the world’s population cannot obtain essential health services and therefore relies on medicinal plants as primary agents to solve their health problems. The complex secondary metabolism of plants produces a variety of specialized metabolites that have provided numerous medicinal compounds used in the modern pharmaceutical industry, particularly in the discovery of new therapeutic agents [24,25]. In fact, plant-derived medicines have been continuously researched for their pharmacological potential against various diseases (e.g., malaria, parasitic diseases, cancer) [25], and, among all Food and Drug Administration (FDA) approvals of new pharmacological entities of natural origin, 25% come from plants [26]. In the context of cancer therapy, plant-derived molecules have exhibited good anti-neoplastic activity with minimal side effects, thus representing an effective, inexpensive, and accessible therapeutic approach [27,28]. In support of this view is the fact that plant-derived anticancer agents, such as the diterpene alkaloid paclitaxel and the indole alkaloids vinblastine and vincristine, are essential drugs used in the treatment of chemotherapy for a wide range of cancer types [29].

**Figure 2:**
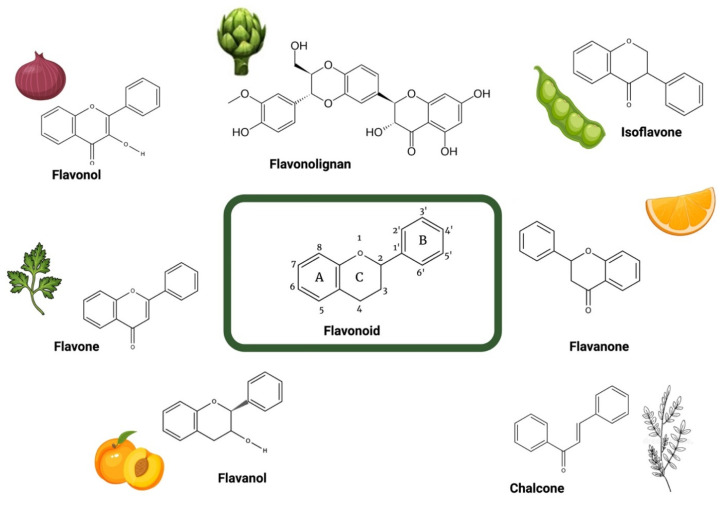
Structure of the different groups of flavonoids reviewed herein with their representative dietary sources. Created with BioRender.com.

Flavonoids are a highly diverse class of specialized metabolites that make up the most important group of polyphenolic compounds present in a plethora of dietary plants, especially in edible fruits, vegetables, and plant-derived beverages (e.g., green tea, wine) [30]. Flavonoids are mainly derived from benzo-γ-pyrone and their basic structure consists of a phenyl ring (ring A) fused with a pyran ring (ring C), in addition to another phenyl ring (ring B) substituted at position 2 of ring C (Figure 2) [30]. The number of different structures already identified belonging to the family of flavonoids exceeds 10,000 [30]. According to the biosynthetic origin and the substitution pattern of the C ring to which the B ring is attached, flavonoids can be classified into subfamilies, mainly flavonols, flavanols, flavones, flavanones, isoflavones, chalcones, and flavonolignans (Figure 2).

Due to their peculiar chemical structure, flavonoids play an essential role in protecting plants from oxidative stress and harmful ultraviolet radiation, as well as from invading microbes [31]. In recent years, a large body of evidence has reported the beneficial effects of flavonoids on various diseases, including cardiovascular diseases, immune-mediated diseases, and cancer [32,33,34,35,36]. In particular, several epidemiological, in vitro, preclinical, and case studies have provided a comprehensive perspective on the anticancer role of flavonoids [37,38,39,40].

## 4. TRAIL-Sensitizing Effects of Flavonoids in Cancer

The studies summarized and discussed here were retrieved from the biomedical literature using “tumor necrosis factor-related apoptosis-inducing ligand” AND “flavonoid” from the PubMed, Scopus, and Web of Science bibliographic databases. The studies were selected after applying the following inclusion and exclusion criteria. The inclusion criteria were (1) papers published in the English language and from 2010 to date; (2) studies investigating naturally occurring flavonoids; and (3) articles with a medium–high citation rate. Exclusion criteria were (1) articles associated with a low credibility rating; (2) articles published in languages other than English; and (3) articles not available in full-text format.

The TRAIL-sensitizing effects of flavonoids in cancer are summarized in Table 1 and discussed below.

### 4.1. Flavones

#### 4.1.1. Apigenin

Apigenin (4′,5,7-trihydroxyflavone) is one of the most studied and widely distributed dietary flavonoids, abundantly found in vegetables (celery, parsley, onions), fruits (oranges), herbs (oregano, thyme, basil), and plant-based beverages such as tea and chamomile infusions [91]. Apigenin has been reported to sensitize non-small cell lung cancer (NSCLC) cell lines A549 and H1299 to TRAIL-induced apoptosis through the c-Jun N-terminal kinase-mediated p53-dependent upregulation of DR4 and DR5 [41]. This effect was also associated with an increase in the pro-apoptotic proteins Bad and Bax and a decrease in the anti-apoptotic proteins Bcl-xl and Bcl-2. The treatment of cells with small molecules specifically inhibiting nuclear factor kappa B (NF-κB), extracellular signal-regulated kinase (ERK) and AKT mirrored the pro-apoptotic effects of apigenin, suggesting a role for these pathways in the TRAIL-sensitizing action of the flavonoid [41]. In particular, the combination of apigenin + TRAIL completely suppressed the growth of A549 cell-derived xenografts in athymic nude mice compared to apigenin or TRAIL monotherapy. Voss and colleagues recently confirmed the ability of apigenin to overcome resistance to TRAIL in lung cancer cells. The authors showed that, by interacting with the RNA-binding proteins hnRNPA2 and MSI2, apigenin reprogrammed the alternative splicing of DR5 and c-FLIP, resulting in increased levels of DR5 and decreased levels of the c-FLIP protein [42]. Oishi et al. and Kim et al. also reported a role for DR5 in apigenin-mediated TRAIL sensitization in prostate cancer cells (DU145 and LNCaP) and hepatocellular carcinoma (HCC) cells (HepG2), respectively [43,44]. Mechanistically, the authors associated the apigenin-driven enhancement of TRAIL-induced apoptosis with the post-transcriptional upregulation of DR5 after the binding and inhibition of adenine nucleotide translocase-2 (ANT2) [43] and with the ERK-induced upregulation of the DR [44]. Regarding HCC, in 2018, Kang and co-workers corroborated the ability of apigenin to enhance TRAIL-induced apoptosis in both the Hep3B and HepG2 cell lines, linking these effects with a reactive oxygen species (ROS)-independent increase in DR5 levels [45].

#### 4.1.2. Luteolin

Luteolin (3′,4′,5,7-tetrahydroxyflavone) is a common flavonoid abundant in vegetables (such as green peppers, celery, parsley, broccoli, cabbages) and fruits (lemon, apple skins). Using an NSCLC xenograft mouse model, Yan et al. demonstrated an augmented antitumor effect of TRAIL in combination with luteolin compared to single treatments, determined by a decreased tumor volume and an increased fraction of TUNEL-positive cells [46]. Luteolin enhanced the TRAIL sensitivity of NSCLC cells (A549 and H1975) but not of the human normal lung epithelial cell line Beas-2B through a mechanism involving the upregulation of DR5 expression and dynamin-related protein 1 (Drp1)-dependent mitochondrial fission [49]. Furthermore, luteolin treatment alone or in combination with TRAIL increased the phosphorylation of c-Jun N-terminal kinase (JNK), while SP600125 (the JNK inhibitor) significantly abolished the synergistic effect on DR5 expression and Drp1 translocation, indicating that the activation of JNK signaling was closely associated with the synergistic effect exerted by luteolin in NSCLC cells [49]. Ou and colleagues showed a positive effect of luteolin on TRAIL sensitivity in the human renal cell carcinoma (RCC) cell lines 786-O, ACHN, and A498 [47]. The cotreatment of 786-O cells with TRAIL and non-toxic concentrations of luteolin resulted in a marked reduction in cell viability, compared to cells treated with TRAIL or luteolin alone. This effect was accompanied by Bid cleavage, Mcl-1 and c-FLIP downregulation (secondary to AKT and signal transducer and activator of transcription 3 (STAT3) inhibition), DR4 and DR5 cell surface presentation, and caspase-8 and caspase-9 activation, suggesting the involvement of both the extrinsic and intrinsic apoptotic pathways in the luteolin-driven TRAIL sensitization [47]. Finally, Nazim et al. reported that cotreatment with luteolin and TRAIL markedly initiated the death of Huh7 and Hep3B HCC cells, which are highly resistant to treatment with luteolin or TRAIL alone, through a mechanism that involved the induction of autophagy and JNK-mediated expression of DR5 [48].

#### 4.1.3. Chrysin

Chrysin (5,7-dihydroxyflavone) is a flavone bearing anti-inflammatory, antioxidant, hepatoprotective, and anticancer effects, typically found in honey, propolis, carrots, chamomile, many fruits, and mushrooms. Ding and co-workers showed that chrysin could break TRAIL resistance in human T cell leukemia virus type 1 (HTLV-1)-associated adult T cell leukemia/lymphoma (ATL) cells by downregulating c-FLIP and by increasing DR5 RNA transcripts [53]. The authors also reported that chrysin improved TRAIL-mediated apoptosis in other human cancer cell lines, including breast cancer (MDA-MB-231), CRC (HT-29), HCC (HepG2), melanoma (SK-MEL-37), and pancreatic carcinoma (Capan-1) cell lines, by the same mechanism [53]. The TRAIL sensitization effects of chrysin have also been reported in A549 and HeLa cells and associated with the inhibition of STAT3 activation and subsequent downregulation of Mcl-1 gene expression. A decrease in Mcl-1 promoted the release of mitochondrial cytochrome c induced by tBid, leading to caspase-9 activation, which was synergized with caspase-8 to activate caspase-3 and PARP cleavage [52]. Subtoxic concentrations of chrysin selectively enhanced TRAIL-induced apoptosis in HCC cells (Hep3B, Huh-7, and Hep G2) but not in human embryo liver L-02 cells or normal human peripheral blood mononuclear cells (PBMCs), by endoplasmic reticulum (ER) stress-dependent CCAAT enhancer-binding protein homologous protein (CHOP)-mediated DR5 upregulation [51]. Finally, Zhang et al. showed that combined treatment with chrysin and TRAIL induced a strong apoptotic response in HepG2 cells, acute leukemia Jurkat T cells, and HeLa cells [50]. Chrysin upregulated the expression of the pro-apoptotic protein Bax and attenuated the expression of anti-apoptotic proteins Bcl-2, Mcl-1, and IAPs. Furthermore, chrysin was well tolerated in mice and synergized with TRAIL in the treatment of HepG2-derived tumor xenografts in vivo [50].

#### 4.1.4. Other Flavones

In addition to apigenin, luteolin, and chrysin, other flavones have been reported to exert TRAIL-sensitizing effects in different types of cancer [54].

Concerning NSCLC, baicalin (5,6-dihydroxy-7-o-glucuronide flavone), a flavone derivative isolated and purified from the root of the Chinese medicinal herb *Scutellaria baicalensis Georgi*, was able to sensitize A549 and H2009 cells to TRAIL-induced apoptosis via p38 MAPK activation and ROS accumulation [55]. Non-toxic concentrations of linarin (a glycosylated flavone identified from various plant species mainly belonging to the Asteraceae and Lamiaceae families) and liquiritin (a major constituent of *Glycyrrhiza Radix*) significantly potentiated the TRAIL-driven cell death of human malignant glioma cells (U87MG) and gastric adenocarcinoma cell lines (AGS and SNU-216, IC50: 79.56 and 78.69 µM respectively), in vitro and in vivo, through ROS generation and the activation of intrinsic and extrinsic apoptotic pathways [56,57]. Similar results have been reported in TRAIL-resistant androgen-dependent LNCaP and androgen-independent DU145 and PC3 prostate cancer cells stimulated with fisetin (3,3′,4′,7-tetrahydroxyflavone), a natural flavonol abundantly found in apple, strawberry, grape, kiwi fruit, persimmon, cucumber, and onion [58]. Finally, galangin (3,5,7-trihydroxyflavone), abundant in propolis and in the rhizome of *Alpinia officinarum*, potentiated TRAIL-mediated apoptosis in TRAIL-resistant renal carcinoma Caki cells and in human breast cancer cell lines (MCF-7 and T47D) by affecting the expression of Bcl-2, c-FLIP, Mcl-1, and survivin at post-translational levels, and the AMPK signaling pathway, respectively [59,60].

### 4.2. Flavonols

#### 4.2.1. Quercetin

Quercetin (3,3′4′,5,7-pentahydroxyflavone) is found in many fruits, vegetables, leaves, seeds, and grains, representing one of the most abundant dietary flavonoids. In particular, capers, red onions, and kale are rich in this flavonol. The TRAIL-sensitizing effects of quercetin have been reported in a variety of cancer types. Combining quercetin with TRAIL treatments may be useful in the treatment of non-Hodgkin’s lymphoma, as suggested by Jacquemin and co-workers [61]. In detail, quercetin restored TRAIL-induced cell death in resistant transformed follicular lymphoma B-cell lines (VAL, RL, and SUDHL4), despite high levels of Bcl-2 due to the chromosomal translocation t(14;18). Quercetin rescued mitochondrial activation by inducing the proteasomal degradation of Mcl-1 and by inhibiting survivin expression at the mRNA level, regardless of p53. Restoration of the TRAIL pathway required Bax and Bak, but it was independent of enhanced TRAIL DISC formation. Yi et al. showed that quercetin enhanced the apoptotic death of ovarian cancer cells (SKOV-3, OVCAR-3, and TOV-21G) to TRAIL (IC50 values for quercetin: SKOV-3: 153.3 ± 4.03 µM, OVCAR-3: 147.4 ± 3.86 µM, and TOV-21G: 159.4 ± 3.64 µM) via the upregulation of the CHOP-induced expression of DR5 after ROS-mediated endoplasmic reticulum stress. Consistent with in vitro findings, quercetin enhanced the TRAIL-mediated inhibition of the tumor growth of a human SKOV-3 xenograft and this effect was associated with the induction of apoptosis and activation of caspase-3, CHOP, and DR5 [63]. Quercetin was able to sensitize TRAIL-resistant pancreatic cancer (8988 T) and breast cancer cell lines (BT-20 and MCF-7 cells) to TRAIL-induced apoptosis These effects were associated with the reduced expression of the c-FLIP protein, resulting from JNK-mediated protein degradation, as well as the transcriptional upregulation of DR5, respectively [62,64]. Finally, Turner et al. showed that TRAIL-resistant malignant melanoma cell lines (WM164 and MeWo) were sensitized by quercetin through the upregulation of DR4 and DR5 on the surfaces of cancer cells and by an increased rate of the proteasome-mediated degradation of c-FLIP [65].

#### 4.2.2. Kaempferol

Kaempferol (3,4′,5,7-tetrahydroxyflavone) is found in a variety of plants and plant-derived foods, including kale, aloe vera, grapes, tomatoes, tea, potatoes, beans, spinach, and broccoli. Kaempferol sensitized ovarian cancer cells (OVCAR-3 SKOV-3) to TRAIL-induced apoptosis via the upregulation of DR4 and DR5 through the ERK/JNK/CHOP pathways [66]. The acute lymphoblastic leukemia MOLT-4 cell line is one of the most resistant cell lines to TRAIL and developed resistance to TRAIL through different pathways. Hassanzadeh et al. found that kaempferol could inhibit the expression of c-FLIP, XIAP, cIAP1/2, FGF-8, and VEGF-beta and conversely augment the expression of DR4/5 in MOLT-4 cells, thus overcoming their resistance to TRAIL [67].

#### 4.2.3. Other Flavonols

In addition to quercetin and kaempferol, TRAIL-sensitizing properties have been reported for flavonols casticin, amurensin, and isoquercitrin in colorectal and cervical cancers. In detail, casticin, a flavonoid isolated from *Vitex rotundifolia* and widely used as an anti-inflammatory agent in Chinese traditional medicine, was found to enhance the TRAIL-induced apoptosis of CRC cells (HT-29, HCT-116, SW480) through the downregulation of cell survival proteins (Bcl-xL, Bcl-2, survivin, XIAP, and c-FLIP) and the ROS-mediated induction of DR5 [68]. In the same context, Lee et al. reported a role for amurensin G, a flavonol isolated from the stem of *Vitis amurensis*, in increasing the sensitivity of cancer stem cell-enriched HCT-15 colony cells. The authors showed that HCT-15 cells positive for CD44, a marker for cancer stem cells from many solid malignancies, were more susceptible to TRAIL-mediated cytotoxicity than CD44− HCT-15 cells, possibly due to increased levels of death receptors DR4 and DR5, as well as c-Myc, and decreased levels of c-FLIP in CD44+ cells compared to CD44− HCT-15 cells. The combination effect of amurensin G on TRAIL-mediated cytotoxicity was much more evident in CD44+ cells than in CD44− HCT-15 cells, and this was associated with the more prominent downregulation of c-FLIP in CD44+ cells than in CD44− HCT-15 cells [69]. More recently, the combination of isoquercitrin, an active metabolite isolated from the leaves of the mangrove tree *Avicennia marina*, and/or rhTRAIL was shown to markedly increase DR4 and DR5 expression in the cervical cancer cell line SiHa, although the increase in the percentage of apoptosis was exiguous, probably due to the presence of other anti-apoptotic proteins [70].

### 4.3. Isoflavones

Isoflavones are substituted derivatives of isoflavone, a type of naturally occurring isoflavonoid. The main sources of isoflavones are plants of the Fabaceae family (Leguminosae), such as soybean (*Glycine max*), red clover (*Trifolium pratense*), white clover (*Trifolium repens*), and alfalfa (*Medicago sativa*).

Genistein, a major isoflavone compound in soybeans and soy products, has been reported to enhance TRAIL-induced apoptosis in the endometrial cell line Ishikawa and in TRAIL-resistant A549 human lung adenocarcinoma cells by unleashing the death receptor signaling pathway (increase in DR4 and DR5, reduction in c-FLIP) and by inhibiting autophagic flux, respectively [71,72]. Biochanin-A, a dietary isoflavone found in soy and red clover, was reported to overcome resistance to TRAIL in the prostate cancer cell lines LNCaP and DU145. In the former, this effect was associated with the increased expression of DR5 and the disruption of mitochondrial membrane potential [73]. The cotreatment of TRAIL and neobavaisoflavone, an isoflavone isolated from *Psoralea corylifolia*, synergistically sensitized U373MG glioma cells to TRAIL-mediated apoptosis via the upregulation of DR5 expression. This sensitization might be associated with Bax induction and Bid truncation, leading to caspase-dependent mitochondrial apoptosis and the inhibition of migration and invasion in U373MG cells [74].

More recently, Xu et al. investigated the effects of irigenin, an isoflavonoid isolated from the rhizome of *Belamcanda chinensis*, in overcoming the resistance to TRAIL of gastric adenocarcinoma cells. Irigenin alone and TRAIL alone did not show an effective role in the induction of apoptosis, whereas their combined treatment significantly induced apoptosis in SGC-7901 cells, as evidenced by the upregulation of cleaved caspase-8/-9/-3 and PARP, in an ROS-dependent fashion. TRAIL sensitization was accompanied by enhanced pro-apoptotic proteins, including FADD, DR5, and Bax, and by a decrease in c-FLIP, Bcl-2, and survivin. Finally, the combination of irigenin and TRAIL significantly inhibited the growth of SGC-7901-derived tumor xenografts in nude mice [75].

### 4.4. Other Flavonoids

Although less investigated, compounds belonging to other families of flavonoids have been reported to possess TRAIL sensitization properties. Chalcones (1,3-diphenyl-2-propen-1-ones) represent an important group of flavonoids widely distributed in various spices, fruits, vegetables, and tea or beer and exhibiting broad anticancer activity through multiple mechanisms [92]. Szliszka and co-workers examined the cytotoxic and apoptotic effects of TRAIL in combination with chalcones isobavachalcone and licochalcone A in cervical cancer HeLa cells, presenting reduced expression of DR that was associated with TRAIL resistance. Both chalcones tested enhanced TRAIL-induced apoptosis in HeLa cells by increasing DR5 expression [76]. Kauntz et al. investigated the effect of silibinin, a flavonolignan that is the main active component of the milk thistle plant (*Silybum marianum*), and TRAIL in an in vitro model of human colon cancer progression, consisting of primary colon tumor cells (SW480) and their derived TRAIL-resistant metastatic cells (SW620). Silibinin sensitized both cell lines to TRAIL-induced apoptosis through the upregulation of DR (DR4 and especially DR5) and activation of extrinsic and intrinsic apoptotic pathways. Additional mechanisms underlying the synergistic effects between silibinin and TRAIL included the downregulation of the Mcl-1 and XIAP proteins [77]. A combinatorial treatment approach with silibinin and TRAIL was also considered to sensitize TRAIL-resistant TNBCs. Manouchehri and colleagues found that silibinin was effective in overcoming the challenge of TRAIL resistance in TNBC BT-20 and HCC1937 cells through the upregulation of DR and downregulation of survivin at the transcriptional level [93]. Regarding the flavanol family, epigallocatechin-3-gallate (EGCG), a major constituent of green tea with anti-diabetes, anti-obesity, anti-inflammatory, and antitumor effects, sensitized human 786-O renal cell carcinoma cells to TRAIL-induced apoptosis by the downregulation of c-FLIP, Mcl-1, and Bcl-2 [78]. More recently, EGCG has been found to be a potent TRAIL sensitizer via the upregulation of DR5 and activation of the extrinsic apoptotic pathway in colorectal cancer cells (SW480 and HCT116) [79]. Finally, combination therapy with naringenin, a natural antioxidant flavanone isolated from citrus fruits, and TRAIL resumed the sensitivity of cultured glioma cells (U251-MG and LNZ308) to TRAIL-driven apoptosis without detectable toxic effects on normal cells of the central nervous system. This effect was associated with the increased expression of DR, upregulation of the pro-apoptotic factors Bad and Bak, and downregulation of Bcl-2 and Bcl-xL. Furthermore, in a mouse xenograft model, cotreatment with naringenin and TRAIL markedly suppressed U251-MG-derived glioma growth by activating apoptosis in tumor tissues, compared to naringenin or TRAIL monotherapy [80].

### 4.5. Prenylflavonoids

Prenylated flavonoids or prenylflavonoids are known to possess phytoestrogenic or antioxidant properties and are widely distributed throughout the plant kingdom. Chemically, prenylflavonoids are characterized by a prenyl group attached to their flavonoid backbone, which is assumed to facilitate attachment to cell membranes and, consequently, the activity of their original flavonoid.

In the last decade, accumulating evidence has pinpointed the TRAIL-sensitizing properties of prenylflavonoids in different types of cancer. Regarding brain cancer, TRAIL-sensitizing effects have been reported for icaritin and morusin in glioblastoma and for xanthohumol in neuroblastoma. Han and colleagues assessed the effect of icaritin, a hydrolytic product of icariin from *Epimedium Genus*, on TRAIL sensitivity in human glioblastoma U87 and U373 cells. The authors found that the non-toxic concentration of icaritin alone had no significant effect on the level of apoptosis, but a combination treatment of TRAIL and icaritin caused markedly increased apoptosis, accompanied by the downregulation of c-FLIP and inhibition of NF-κB activity. Of note, NF-κB knockdown by shRNA enhanced apoptosis in TRAIL-treated U87 and U373 cells, similar to icaritin [81]. Similarly, Park et al. showed that the combination treatment of TRAIL with morusin, an active metabolite isolated from the root bark of *Morus alba* L., synergistically increased apoptosis compared to single agents in glioblastoma cell lines. Mechanistically, morusin treatment induced the expression of DR5, but not DR4 or DcR1 and DcR2, and decreased the protein levels of survivin and XIAP, possibly by reducing the expression of epidermal growth factor receptor and platelet-derived growth factor receptor, as well as STAT3 phosphorylation/activation [82]. Finally, prenylated chalcone xanthohumol in combination with TRAIL was shown to significantly increase DR5 expression and the percentage of apoptosis in human cervical cancer (HeLa) and neuroblastoma (NGP, SH-SY-5Y, and SK-N-AS) cell lines compared to TRAIL or xanthohumol alone [76,83].

Several prenylflavonoids have shown TRAIL-sensitizing properties against gastric adenocarcinoma cells. Heterophyllin enhanced the expression of DR4 and DR5 transcripts (presumably in a CHOP-dependent manner) and increased caspase-3/-7 activity when combined with TRAIL in AGS cells [84]. In the same cells, artonin E increased caspase-3/-7 activity when used in combination with TRAIL through the ROS- and p53-mediated upregulation of DR5 protein levels [85], whereas kurarinone promoted TRAIL-induced apoptosis in SGC7901 gastric adenocarcinoma cells by inhibiting Mcl-1 and c-FLIP expression by modulating STAT3 [86]. The synergistic effects of kurarinone and TRAIL in inducing apoptosis were also observed in cervical cancer cells (HeLa) and associated with the NF-κB-dependent suppression of c-FLIP expression [87].

Other prenylated flavonoids have demonstrated TRAIL-sensitizing actions in cells from melanoma, prostate cancer, and colorectal cancer. Du et al. showed that icariside II, an active component of *Herba Epimedii*, could potentiate TRAIL-driven cell death in melanoma A375 cells, presenting a low response to the pro-apoptotic action of the cytokine, through the ROS-mediated downregulation of STAT3/c-FLIP signaling. The results were confirmed in TRAIL-resistant melanoma cells (MeWo and SK-MEL-28), which were converted into TRAIL-sensitive cells by icariside II treatment [88]. Combined treatment with auriculasin, a prenylated isoflavone found in various food ingredients, such as the roots of *Flemingia philippinensis*, the stem bark of *Erythrina senegalensis*, and osage orange fruits, and TRAIL resulted in tumor-specific apoptotic cell death in RC-58T/h/SA#4 primary prostate cancer cells, characterized by DNA fragmentation, the accumulation of apoptotic cell populations, and nuclear condensation. This effect was correlated with the inhibition of DR5, CHOP, and p53 expression [89]. Finally, Kim et al. reported that icariin, a prenylated flavonol glycoside derived from the Chinese herb *Epimedium sagittatum*, increased the effects of TRAIL to induce apoptosis in the CRC cell line HCT-116 in vitro and to reduce the in vivo growth of HCT-116-derived tumors in a xenograft mouse model via the ROS-, ERK-, and CHOP-mediated upregulation of DR5 and DR4 [90].

## 5. Discussion

The immune cytokine TRAIL has attracted significant attention in oncology due to its ability to selectively eliminate malignant cells in a wide range of cancers, without inciting toxicity in normal cells [11]. However, despite its tremendous potential for cancer therapy, both in vitro and in vivo studies have shown that a non-negligible proportion of cancer cell lines harbor innate resistance to TRAIL, which can occur at virtually every step of the cell signaling cascade [19]. Furthermore, as the tumor genome evolves under selective pressure, a large number of tumors can also acquire resistance to TRAIL at later stages [20]. These hurdles have limited the translation of TRAIL-based therapies into the clinic and highlighted the importance of developing strategies to overcome TRAIL resistance. In this regard, accumulating studies have shown that TRAIL-mediated apoptosis can be facilitated in resistant tumors by combined treatment with antitumor agents, ranging from synthetic molecules to natural products [94]. Among the latter, flavonoids, the most prevalent polyphenols in plants, have shown remarkable competence to improve TRAIL-driven apoptosis in resistant cell lines and in tumor-bearing mice.

**Figure 3:**
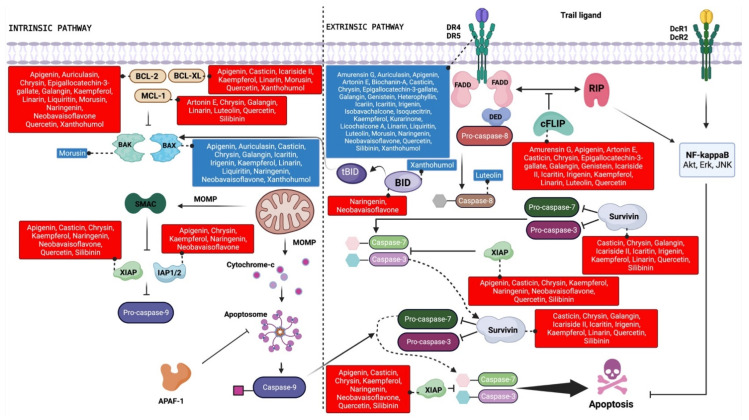
Schematic representation of the TRAIL signaling pathway indicating the therapeutic targets modulated by flavonoid compounds. The blue and red boxes indicate upregulation and downregulation, respectively, of the target protein by the indicated flavonoids. Created with BioRender.com.

Although the exact mechanisms that contribute to the escape from TRAIL-induced apoptosis and the progress of resistance to TRAIL in tumor cells have not yet been fully unraveled, downregulating pro-apoptotic proteins and DR4/5, concomitant with modulating intracellular pro- and anti-apoptotic proteins, would seem to be of paramount importance [19]. Almost all flavonoids reviewed here can increase the susceptibility to TRAIL-induced apoptosis in part or completely by the upregulation of DR4 and/or DR5 (Table 1; Figure 3).

These findings are interesting because recombinant human TRAIL or TRAIL receptor agonists administered in Phase I and II clinical trials were not only well tolerated, but also promoted prolonged cancer stability [95,96]. As TRAIL receptor agonists have been and are currently used in combination with cytotoxic agents (e.g., Carboplatin, Paclitaxel, Gemcitabine) [97], it is tempting to speculate that combination therapies with selected flavonoids could potentially diminish the risk and severity of chemotherapy-related adverse effects.

Anti-apoptotic proteins such as c-FLIP and IAP and Bcl-2 family proteins play a fundamental role in TRAIL resistance in cancer cells, as they attenuate the sensitivity of these cells to TRAIL through several mechanisms [11]. It should be noted that different classes of flavonoids have shown great potential to increase TRAIL-induced apoptosis through the negative regulation of such molecules (Table 1; Figure 3). Studies have shown that the silencing of c-FLIP restores the apoptotic process and is an efficient method to increase tumor cell sensitivity to TRAIL [98,99]. It is noteworthy that the TRAIL-sensitizing effects observed in a variety of cancer cell lines after combinatory treatment with different flavonoids (i.e., apigenin, luteolin, chrysin, linarin, galangin, quercetin, kaempferol, casticin, amurensin G, genistein, irigenin, epigallocatechin-3-gallate, icaritin, kurarinone, and icariside II) were associated with the decreased expression of c-FLIP (Table 1; Figure 3). Anti-apoptotic proteins belonging to the IAP family share one to three common structures called baculoviral IAP repeat (BIR) domains that enable them to bind to caspases and other proteins, interfering with both extrinsic and intrinsic apoptotic signaling [100]. Many flavonoids with TRAIL-sensitizing effects downregulated IAP proteins (Table 1; Figure 3). Among these, some showed inhibitory action on XIAP (i.e., apigenin, chrysin, quercetin, kaempferol, casticin, neobavaisoflavone, silibinin, naringenin) and survivin (i.e., chrysin, linarin, galangin, quercetin, kaempferol, casticin, irigenin, silibinin, icaritin, icariside II), which are overexpressed in a variety of human cancers and are considered the most potent members of the family [101,102].

Pro- and anti-apoptotic proteins belonging to the Bcl-2 family, structurally differentiated by the BH3 domain, play an essential role in the balance between cell death and cell survival, as well as in the regulation of TRAIL-induced cytotoxicity [11,103]. Given this, the members of the Bcl-2 family are considered attractive targets for the development of agents for the treatment of cancer [104]. In general, agents belonging to all classes of flavonoids reviewed here acted by overcoming TRAIL resistance by modulating the expression of at least one protein of the Bcl-2 family (Table 1; Figure 3).

Aberrant levels of proteins conferring resistance to TRAIL in a variety of cancer cells rely at least in part on the activation of anti-apoptotic signal transduction pathways, such as mitogen-activated protein kinase (MAPK), NF-κB, and STAT3 [105]. In this regard, some of the TRAIL-sensitizing effects mediated by flavonoids have been reported to be secondary to MAPK regulation (as in the case of baicalin, luteolin, quercetin, kaempferol, icariin, and apigenin), STAT3 (kurarinone and icariside II), and NF-κB (kurarinone and icaritin). However, it should be noted that the articles mentioned in this review have not characterized the dose–response relationship between the investigated flavonoids and their TRAIL sensitization properties, thus making it difficult to evaluate the relevance of the reported studies.

As mentioned above, the mechanisms underlying the TRAIL-sensitizing activity of flavonoids are multifaceted, often involving the modulation of different oncogenic pathways/proteins. Thus, it is tempting to speculate that flavonoids that simultaneously act on multiple proteins of the TRAIL cascade may be in a more advantageous position in terms of efficacy than those acting on single signaling checkpoints.

In view of the putative low toxicity displayed by all flavonoids, along with their great anti-cancer potential demonstrated in experimental and preclinical settings, researchers have been trying to explore them clinically. However, it is worth mentioning that, especially when administered at exorbitant amounts, flavonoid intake may result in adverse effects, even severe. The toxicity associated with flavonoids arises from their ability to act as pro-oxidants, modulate cytochrome P450 enzyme activity, interfere with thyroid hormone production, and interact with nuclear estrogen receptors (ER) and aryl hydrocarbon receptor (AHR), among others [106]. Flavonoid-derived toxicity comprises modulatory effects on DNA and carcinogenicity, hepatotoxicity and nephrotoxicity, and effects on the thyroid and reproductive function, as well as intestinal flora disorders [38]. For an overview of the possible toxic side effects of individual flavonoids, we direct the reader to specific reviews that comprehensively address this issue [107,108].

Although, so far, none of the reported flavonoids have been studied in clinical settings for their TRAIL-sensitizing ability, some clinical trials have evaluated the anti-cancer effects of specific flavonoids (e.g., epigallocatechin-3-gallate, quercetin, apigenin, genistein, fisetin, and xanthohumol), showing more positive outcomes for hematopoietic and lymphoid tissues than for solid tumors [109,110,111]. However, these studies do not provide compelling evidence and, to the best of our knowledge, neither the United States Food and Drug Administration (FDA) nor the European Food Safety Authority (EFSA) has approved any flavonoids as prescription drugs so far.

These disappointing results could be due to the bioavailability-related issues (i.e., low solubility and stability, easy degradation by the microbiota and in an extremely acidic medium, low intestinal permeability, high metabolism) characterizing almost all groups of flavonoids under physiological conditions [112]. In fact, one of the most concerning issues that arises when considering the incorporation of flavonoids as therapeutic drugs is their bioavailability and absorption after oral ingestion. In the intestine, flavonoids are generally absorbed through two mechanisms [113]. The first involves an initial step of hydrolysis by a brush border enzyme called lactase phlorizin hydrolase. This leads to the transformation of flavonoids into their aglycone forms, which can readily diffuse into intestinal epithelial cells as a result of their lipophilic properties. The second mechanism starts with the transport of the hydrophilic glycoside form through membrane transporters such as sodium-dependent glucose transporter 1. In this scenario, the hydrolysis step into the aglycone form occurs inside epithelial cells through the action of cytosolic β-glucosidase [113]. Regardless of the mechanism through which they are obtained, the aglycones then undergo selected modifications, such as methylation, sulfation, or glucuronidation. Some of the resulting compounds enter the bloodstream, while others leak back into the intestinal lumen, further lowering the bioavailability of flavonoids. Even the molecules that pass into the bloodstream enter the hepatic circulation, undergo further metabolism, and can be excreted with bile [113]. Therefore, the low bioavailability of flavonoids has been one of the main obstacles that hinders their official introduction into the world of pharmacotherapeutics, despite their promising effects elucidated through in vivo and in vitro experiments [38]. For a detailed analysis of the pharmacokinetics and -dynamics of the different groups of flavonoids reviewed here, we direct the reader to specific reviews that address these issues in a comprehensive way [38,107,114].

To overcome the bioavailability-related drawbacks, several approaches are currently being developed with respect to flavonoid delivery methods, such as nanoparticle-based functionalization (e.g., with specific ligands such as folic acid to confer target skills) [115] and/or encapsulation, with the aim of creating systems that allow the use of lower concentrations of flavonoids and ensure a suitable stability, solubility, absorption, and delivery profile to cancer cells [112]. In particular, encapsulation methods include (i) lipid-based delivery systems (e.g., liposomes, lipid-based nanoparticles, emulsions, and nanoemulsions); (ii) polymer-based nanoparticles (encompassing natural, synthetic, and inorganic polymers); (iii) micelles, which are constituted by amphiphilic molecules; (iv) inclusion complexes, defined as delivery systems characterized by having a host molecule with the ability to trap another molecule using non-covalent forces; and (v) dendrimers, which are made up of polymeric materials that have a highly branched architecture with numerous functional groups and an interior cavity that allows drug encapsulation [112]. Despite the undoubted therapeutic superiority over free flavonoids, all of these delivery systems carry challenges. For instance, nanocarriers have to face both formulation and stability issues, as well as government regulations and higher costs; inclusion complexes possess a limited encapsulation rate for larger flavonoids, such as glycosylated compounds; and dendrimers may present toxicity concerns. For a detailed overview of this topic, we direct the reader to recent state-of-the-art reviews [112,115,116,117].

The above-mentioned flavonoid delivery systems have been optimized and tested in cancer therapies in both cultured cells and experimental models with encouraging outcomes, resulting in a relevant contribution to progress towards the design/conception of flavonoid-based carriers [112]. However, clinical studies are needed and are actually underway to address whether these new formulations aimed at increasing the bioavailability of flavonoids could improve the success rates of these compounds as auxiliary therapies in the treatment or prevention of various types of cancer.

## 6. Conclusions

Taken together, the studies described and discussed in this review suggest a promising role for some flavonoids as adjuvant agents in TRAIL-based therapies. However, most, if not all, of the above-mentioned shortcomings should be addressed to increase the probability of success in the clinic.

## Figures and Tables

**Table 1:** Molecular mechanisms underlying the TRAIL-sensitizing properties of different flavonoids in cancer cells.

Compound (Class)	Cancer Type	Mechanism(s) of Action	Concentration/s	Reference
Apigenin **(Flavone)**	NSCLC	↑Bax, ↑Bad, ↑DR4/5, ↓Bcl-2, ↓Bcl-xL, ↓c-FLIP	10–40 µM	[41]
	NSCLC, primary patient-derived lung cancer cells	↑DR5, ↓c-FLIP	10–50 µM	[42]
	Prostate cancer	↑DR5	5–20 µmol/L	[43]
	HCC	↑DR4/5	2–30 µg/mL	[44]
	HCC	↑DR5, ↓Bcl-2, ↓IAP-1/2, ↓XIAP	15–20 µM	[45]
Luteolin **(Flavone)**	Lung cancer, cervical cancer	↑Caspase-8	5 µM	[46]
	RCC	↑DR4/5, ↓c-FLIP, ↓Mcl-1	10 µM	[47]
	HCC	↑DR5	5–20 µM	[48]
	NSCLC	↑DR5	5–40 µM	[49]
Chrysin **(Flavone)**	HCC	↑Bax, ↓Bcl-2, ↓IAP-1/2, ↓Mcl-1, ↓Survivin, ↓XIAP	10–40 µM	[50]
	HCC	↑DR5	2.5–10 µmol/L	[51]
	Cervical cancer, lung cancer	↓Mcl-1	60–100 µM	[52]
	CRC, HCC, breast cancer, ATL, melanoma, pancreatic carcinoma	↑DR5, ↓c-FLIP, ↓Mcl-1,	50–100 µM	[53]
7-Hydroxyflavone **(Flavone)**	CRC	-	50–100 µM	[54]
Baicalin (**Flavone**)	Lung cancer	-	50–100 µM	[55]
Liquiritin **(Flavone)**	Gastric adenocarcinoma	↑Bax, ↑DR4/5, ↓Bcl-2	25–200 µM	[56]
Linarin **(Flavone)**	Glioma	↑Bax, ↑DR4/5, ↓Bcl-2, ↓Bcl-xL, ↓c-FLIP, ↓Mcl-1, ↓Survivin	5 µM	[57]
Fisetin (**Flavone**)	Prostate cancer	-	10–50 µM	[58]
Galangin **(Flavone)**	RCC	↓Bcl-2, ↓c-FLIP, ↓Mcl-1, ↓Survivin,	30 µM	[59]
	Breast cancer	↑Bax, ↑DR4, ↓Bcl-2	20–40 µM	[60]
Quercetin **(Flavonol)**	Non-Hodgkin’s B-lymphoma	↓Mcl-1, ↓Survivin	20 µM	[61]
	Pancreatic cancer	↓c-FLIP	30–90 µM	[62]
	Ovarian cancer	↑DR5, ↓Bcl-2, ↓Bcl-xL, ↓Survivin, ↓XIAP	50–200 µM	[63]
	Breast cancer	↑DR5, ↓c-FLIP	12.5–50 µM	[64]
	Melanoma	↑DR4/5, ↓ c-FLIP	25–50 µM	[65]
Kaempferol **(Flavonol)**	Ovarian cancer	↑Bax, ↑DR4/5, ↓Bcl-2, ↓Bcl-xL, ↓c-FLIP, ↓Survivin, ↓XIAP	25–100 µM	[66]
	Acute lymphoblastic leukemia	↑DR4/5, ↓c-FLIP, ↓IAP-1/2, ↓XIAP	95 µM	[67]
Casticin **(Flavonol)**	CRC	↑Bax, ↑DR5, ↓Bcl-xL, ↓c-FLIP, ↓Survivin, ↓XIAP	1–3 µmol/L	[68]
Amurensin G **(Flavonol)**	CRC	↑DR4/5, ↓c-FLIP	1–5 µM	[69]
Isoquecitrin **(Flavonol)**	Cervical cancer	↑DR4/5	100–1400 µM	[70]
Genistein **(Isoflavone)**	Lung cancer	-	10–40 µM	[71]
	Endometrial cancer	↑DR4/5, ↓c-FLIP	10–50 µM	[72]
Biochanin-A **(Isoflavone)**	Prostate cancer	↑DR5	100 µM	[73]
Neobavaisoflavone **(Isoflavone)**	Glioma	↑Bax, ↑DR5 ↓Bid, ↓Bcl-2, ↓IAP-3, ↓XIAP	5–30 µM	[74]
Irigenin **(Isoflavone)**	Gastric adenocarcinoma	↑Bax, ↑DR5, ↓Bcl-2, ↓c-FLIP, ↓Survivin	5–10 µM	[75]
Isobavachalcone **(Chalcone)**	Cervical cancer	↑DR5	25–50 µM	[76]
Licochalcone A **(Chalcone)**	Cervical cancer	↑DR5	25–50 µM	[76]
Silibinin **(Flavonolignan)**	CRC	↑DR4/5, ↓Mcl-1, ↓XIAP	100–300 µM	[77]
	Breast cancer	↑DR4/5, ↓Survivin	25–50 µM	[64]
Epigallocatechin-3-gallate **(Flavanol)**	RCC	↓Bcl-2, ↓c-FLIP, ↓Mcl-1	50 µg/mL	[78]
	CRC	↑DR5	20–60 µM	[79]
Naringenin **(Flavanone)**	Glioma	↑Bax, ↑DR5 ↓Bid, ↓Bcl-2, ↓IAP-3, ↓XIAP	160 µM	[80]
Icaritin **(Prenylflavonoid)**	Glioblastoma	↑Bax, ↑DR5, ↓Bcl-2, ↓c-FLIP, ↓Survivin	10–20 µM	[81]
Morusin **(Prenylflavonoid)**	Glioma	↑Bak, ↑Bad, ↑DR4/5, ↓Bcl-2, ↓Bcl-xL	2.5–5 µM	[82]
Xanthohumol **(Prenylflavonoid)**	Cervical cancer	↑DR5	25–50 µM	[76]
	Neuroblastoma	↑Bax, ↑Bid, ↑DR5 ↓Bcl-2, ↓Bcl-xL	7.5 µM	[83]
Heterophyllin **(Prenylflavonoid)**	Gastric adenocarcinoma	↑DR4/5	4 µM	[84]
Artonin E **(Prenylflavonoid)**	Gastric adenocarcinoma	↑DR5	3 µM	[85]
Kurarinone **(Prenylflavonoid)**	Gastric adenocarcinoma	↓c-FLIP, ↓Mcl-1	5 µM	[86]
	Cervical cancer	↑DR5	5 µM	[87]
Icariside II **(Prenylflavonoid)**	Melanoma	↓Bcl-xL, ↓c-FLIP, ↓Survivin	20 µM	[88]
Auriculasin **(Prenylflavonoid)**	Prostate cancer	↑Bax, ↑DR5, ↓Bcl-2	2.5–10 µM	[89]
Icariin **(Prenylflavonoid)**	CRC	↑DR4/5	1–10 µM	[90]

Abbreviations: B-cell lymphoma 2 protein (Bcl-2); B-cell lymphoma extra-large protein (Bcl-xL); Bcl-2 associated X (Bax); Bcl-2 antagonist/killer 1 (Bak); colorectal cancer (CRC); cellular FLICE-like inhibitory protein (c-FLIP); cellular inhibitor of apoptosis protein (IAP); death receptor 4 (DR4); death receptor 5 (DR5); hepatocellular carcinoma (HCC); non-small cell lung cancer (NSCLC); renal cell carcinoma (RCC); induced myeloid leukemia cell differentiation protein (Mcl-1); pro-apoptotic BH3 interacting-domain death agonist (BID); X-linked inhibitor of apoptosis protein (XIAP). Up arrow indicates upregulation, whereas down arrow indicates downregulation.

## Data Availability

No new data were created or analyzed in this study. Data sharing is not applicable to this article.
